# Impact of Porcine Epidemic Diarrhea on Performance of Growing Pigs

**DOI:** 10.1371/journal.pone.0120532

**Published:** 2015-03-13

**Authors:** Julio Alvarez, Javier Sarradell, Robert Morrison, Andres Perez

**Affiliations:** 1 Department of Veterinary Population Medicine, College of Veterinary Medicine, University of Minnesota, St Paul, Minnesota, United States of America; 2 Department of General and Systemic Pathology (Sarradell), Veterinary Faculty, National University of Rosario, Casilda, Argentina; Cornell University, UNITED STATES

## Abstract

The impact of porcine epidemic diarrhea virus (PEDv) infection on the US pork industry has mainly been attributed to the mortality that it causes in suckling piglets, and, consequently, much effort has been invested in the quantification of its effect in sow farms. However, no information on the performance of surviving pigs that were exposed to the PEDv as piglets is available. Here, a retrospective cohort study to evaluate the impact of porcine epidemic diarrhea virus (PEDv) infection on growing pigs’ performance, as indicated by mortality, average daily gain (ADG), average daily feed intake (ADFI), and feed conversion ratio (FCR) was performed using production records from weaned pigs in nursery and wean-to-finish sites from sow farms that became PEDv-infected between May 2013 and June 2014. Production records from the first batch of growing pigs weaned in infected flows after the PEDv outbreak (“infected batches”) were compared with those from pigs weaned within the previous 14 to 120 days (“control batches”). Performance records from infected and control batches, paired by flow, were compared using non-parametric paired tests. Mortality, ADG and FCR were significantly different in PEDv-positive (infected) compared with PEDv-negative (control) batches, with a mean increase of mortality and FCR of 11% and 0.5, respectively, and a decrease of ADG of 0.16 lb/day. Our results demonstrate a poorer performance of growing pigs weaned after a PEDv outbreak compared with those weaned within the previous 14-120 days, suggesting that in addition to the mortality induced by PEDv in suckling pigs, the disease also impairs the performance of surviving pig. These findings help to quantify the impact of PEDv infection in the US and, ultimately, contribute to efforts to quantify the cost-effectiveness of disease prevention and control measures.

## Introduction

Since its first detection in May 2013[1] Porcine Epidemic Diarrhea virus (PEDv) has caused a major impact on the US pork industry. Pork production is expected to decline around 6–7% in 2014 due to a decrease in the number of hogs that may range from 3 to 11%.[2,3] The devastating effect of PEDv is mainly due to the acute watery diarrhea induced in infected pigs, which may lead to a high (50–80%) mortality, particularly in neonatal piglets.[4] In many sow farms, it may take at least 12 weeks to recover to the baseline piglet production level.[5] However, if piglets survive the first seven days after infection, their chance of survival increase.[6] The impact of PEDv infection in subsequent production stages (i.e., post-weaning/ growing and finishing pigs) of pigs surviving the first contact with PEDv has not been reported.

Performance indicators routinely used to measure pigs’ productivity include average daily gain (ADG), average daily feed intake (ADFI), and feed conversion ratio (FCR).[7] In addition, increased mortality in growing pigs is also related with decreased profitability in swine operations.[8] Here, those four indicators were used to quantify the impact of PEDv infection in growing pigs. These results will help to quantify the impact that the disease has on the swine industry and to estimate the cost-effectiveness of alternative prevention and control measures.

## Material and Methods

### Study population

Data from all batches of weaned pigs were collected from one large swine system between 1^st^ of March 2013 (before PEDv had been reported in the USA) and 1^st^ of June 2014. The database included all batches of animals produced in all farms from the company, located in the Midwest. All sows and boars were cross bred, commercial genetics. All pigs were vaccinated for *Mycoplasma hyopneumoniae*, porcine circovirus (PCV) and porcine reproductive and respiratory syndrome (PRRS). PCV was present in all farms while PRRS virus (PRRSv) was occasionally detected in a proportion of them (see below). Collected data included information on the site in which pigs were located, site production type (nursery or wean to finish (WF)), pig source (sow farm from which pigs originated), start and close period on each site, number of pigs per batch, mortality (defined by percentage of total pigs started), average daily gain (ADG), average daily feed intake (ADFI), feed conversion (FCR), and status of the sow farm from which pigs were weaned for PRRS and PED. Because weaned pigs from different sow farms were sometimes commingled in the subsequent growing stages, the term “flow” is used to refer to the origin of a given pig batch (and may include one or more single sow farms). Only flows in which PED was detected (based on diagnostic investigation including clinical signs and PCR detection of PEDv) during the period of study were selected. The identity of the data provider cannot be disclosed due to legal restrictions, but all relevant information underlying the analyses presented here are contained in the text and S1 Dataset.

### Data analysis

To evaluate the effect of PEDv infection on mortality and performance, a retrospective cohort study was carried out in which the exposed group was the first PED-positive batch from a given flow (infected batch) and the unexposed group (control batch) was defined as the batch that had left the sow farm immediately before the exposed group. Records were screened to identify PED-positive flows, and, for each PED-positive flow, the first PED positive batch (infected group) and the preceding batch (control group), that had to precede the infected group by more than 14 but less than 120 days. For flows with more than one sow farm, only batches with the same combination of sow farms were paired. Candidate control batches leaving sow farms within the first two weeks before the PED-infected batch were excluded from the analysis in an effort to have high specificity for the “control batches”. Target parameters (mortality, ADG, ADFI, and FCR) of infected and control groups were compared using the Wilcoxon test, and differences observed in nursery and WF pairs were compared using a Mann-Whitney test.

## Results

Eighteen flows fulfilled the inclusion criteria, i.e., were positive to PED at some stage during the study period and had a control batch leaving the sow farms within the previous 14–120 days to the first PED-positive batch (S1 Dataset). Of those 18 flows, 4 sent pigs to nurseries (“nursery flows”) and 14 to WF sites (“WF flows”). Only one of the four nursery batches originated from a single sow farm, whereas the other three originated from up to five different sow farms and were commingled in the nursery (“mixed flows”). In contrast, all 14 WF flows originated from one single sow farm (Table 1).

**Table 1:** Performance indicators of 18 pairs of control (before Porcine Epidemic Diarrhea—PED- detection) and infected (first batch after PED detection) pigs going to nursery and wean-to-finish sites.

Production type	Flow	Number of sow farms	Before PED				After PED			
			ADG^a^	FCR^b^	ADFI^c^	Mortality (%)	ADG	FCR	ADFI	Mortality (%)
**Nursery**	A	4	0.65	1.67	1.08	4.60	0.62	1.70	1.06	14.18
	B	2	0.77	1.59	1.23	1.60	0.83	1.92	1.59	24.33
	C	5	0.89	1.71	1.53	9.86	0.83	1.82	1.51	20.63
	D	1	0.84	1.91	1.60	2.60	0.64	2.77	1.77	0.64
**Wean-to-finish**	E	1	0.82	2.15	1.77	1.83	0.68	2.10	1.42	6.38
	F	1	0.87	1.44	1.25	3.61	0.70	2.55	1.78	21.14
	G	1	0.85	1.26	1.07	3.27	0.97	1.99	1.94	10.82
	H	1	0.77	1.48	1.14	2.58	0.80	3.05	2.43	12.52
	I	1	0.90	1.53	1.38	1.76	0.58	2.55	1.48	18.89
	J	1	0.88	1.86	1.64	11.41	0.87	2.04	1.77	17.07
	K	1	0.80	1.81	1.46	6.86	0.35	2.96	1.05	51.67
	L	1	0.69	1.74	1.20	6.91	0.63	2.15	1.36	34.70
	M	1	1.13	1.81	2.04	0.67	0.70	2.28	1.59	7.62
	N	1	0.85	1.75	1.50	1.93	0.77	1.83	1.41	3.30
	O	1	0.79	1.63	1.29	2.65	0.56	1.84	1.02	5.65
	P	1	1.12	1.38	1.55	4.39	0.85	1.59	1.34	5.17
	Q	1	0.97	1.28	1.24	1.76	0.41	2.52	1.04	31.06
	R	1	0.73	1.75	1.27	3.19	0.59	1.93	1.15	9.81

^a^ADG = Average daily gain (lb./day)

^b^ADFI = Average daily feed intake (lb./day)

^c^FCR = Feed conversion ratio

During the four months before the PEDv outbreak, nursery flows produced 23 batches of weaned pigs, with a mean time between batches of 19 days (median = 13 days, range = 3–34 days), whereas 82 batches were produced by the 14 WF flows (between-batch mean and median time of 14 and 15 days, range = 4–63). Median average days on feed was 64 days (interquartile range = 58–69 days) for nursery batches and 66 days for WF batches (IQR = 55–72). Pigs from only 3 of the 82 WF batches weaned before the PED outbreak were on feed for >150 days, with records from the remaining pigs coming from the first stage of a double stocking practice. Those 3 batches were not considered eligible as control/infected batches due to the effect of the longer period on feed on the performance of the pigs. Within the four previous months to the PED outbreak, PRRSv was detected in four of the 18 flows (A, C, E, H) but batches selected as controls from all but one flow (flow C) were already negative. Four case batches were positive for PRRS (flows O, P and Q, in addition to flow C).

**Fig 1:**
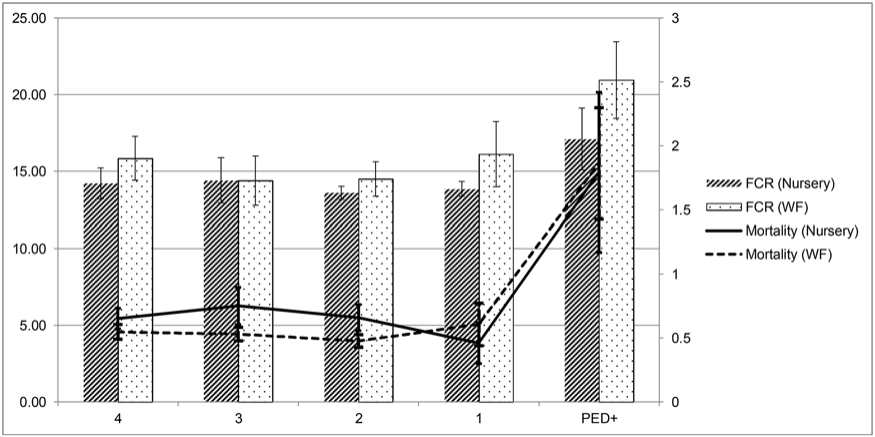
Feed conversion rate (FCR) and mortality in nursery and wean-to-finish batches in the 4 months before Porcine Epidemic Diarrhea detection in 18 flows and in the first batches weaned after the outbreak.

Before the PEDv outbreak overall mean monthly mortality and FCR ranged between 4.3–4.8% and 1.71–1.89 respectively (Fig. 1), whereas ADG and ADFI values were between 0.75–0.85 and 1.29–1.62 (data not shown). Analysis of the mortality of the first PED-positive batches on each flow revealed an increase in the mortality up to 14.9% in nursery and 15.5% in WF (Fig. 1). ADG was lower in WF batches compared with the previous four months, while this effect was not so apparent in nursery pigs, and ADFI observed in the outbreak batches did not increase in WF batches and increased moderately in nursery pigs compared with the values observed in the previous months. As a consequence, a strong increase in the FCR recorded in the infected batches was observed (Fig. 1).

**Fig 2:**
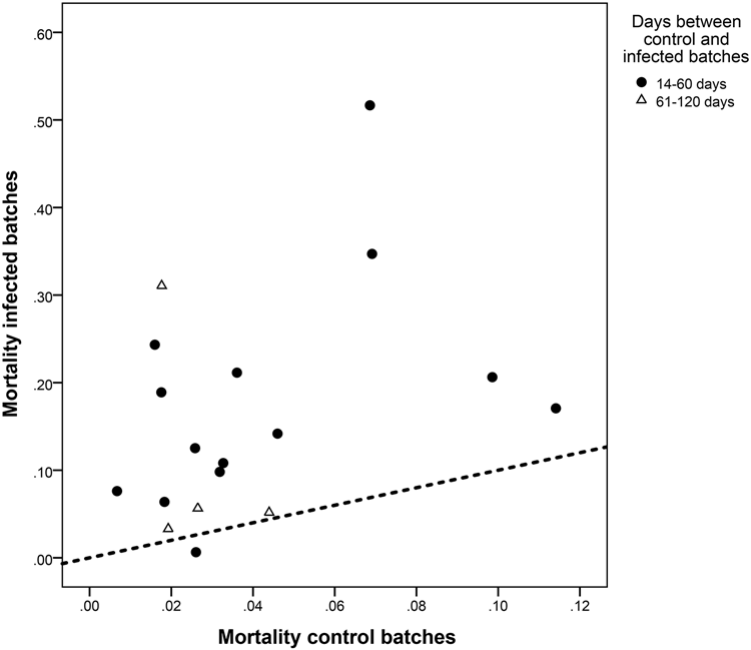
Mortality observed in paired batches from 18 flows in the first batch produced after PED detection in the sow farms (infected batches) and in the immediate previous batch (produced within the previous four months, control batches). Dotted line: no differences in mortality.

Mortality was significantly (p<0.001) higher in PED-positive batches compared with the control pigs, with an overall mean difference of 12.5% (95% confidence interval = 6.4–18.4) (Fig. 2). Difference observed in WF pairs was not significantly different from that found in nursery pairs. The difference in mortality between control and infected batches increased for those cases in which control batches left the sow farms in the previous 14–60 days compared with 61–120 days (Fig. 2).

**Fig 3:**
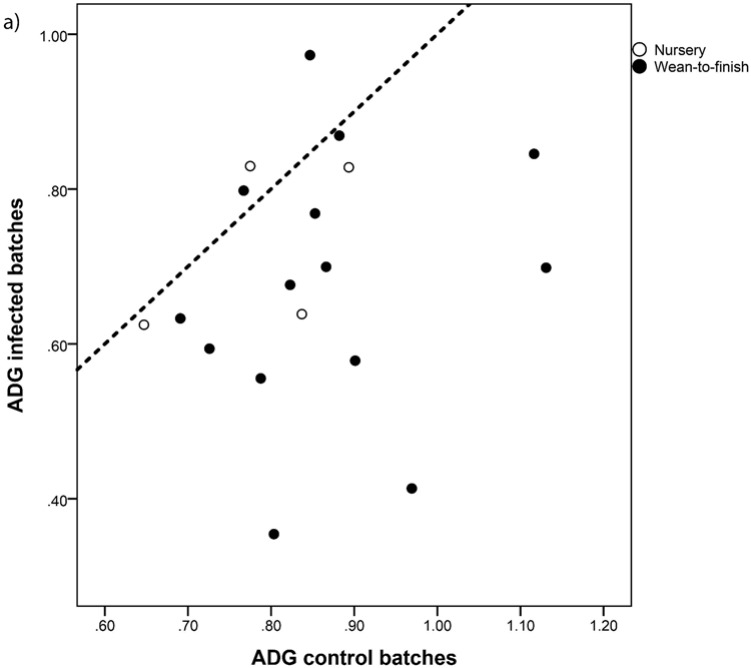
Average daily gain (ADG, Fig. 3a) and feed conversion ratio (FCR, Fig. 3b) observed in paired batches from 18 flows in the first batch produced after PED detection in the sow farms (infected batches) and in the immediate previous batch (produced within the previous four months, control batches). Dotted line: no differences in ADG/FCR.

ADG and FCR were significantly worse in case batches, with a 0.16 lb mean decrease of the former (95% CI = 0.07–0.26) (Fig. 3a) and a 0.55 increase in the latter (95% CI = 0.30–0.79) (Fig. 3b). ADFI remained largely unaffected (p = 0.9). Again, differences observed in WF batches were larger than those observed in nursery pigs, but differences were not significant (p>0.3).

## Discussion

The devastating effect of PEDv in suckling piglets caused a decrease in all production indicators in US sow farms since its first detection in May 2013. However, although mortality in piglets may be as high as 80% in the first days of the outbreak, sow farms may return to their baseline production within 13 weeks, although reaching 100% of the baseline production may take longer [5]. In addition, PEDv infection may have an effect in surviving piglets that may impair their future performance and, therefore, increase the economic burden caused by PEDv infection; still, to the authors’ knowledge, this hypothesis has not been tested yet. In the present study we compared the performance of pigs produced in 18 different flows before and just after diagnosis of PED in the sow farms in a first attempt to detect the impact of PEDv infection in growing pigs.

Comparison of the mean mortality rates observed in the batches produced in the four months before the PEDv reveals a rather stable trend with point estimates usually below 6%, and with 95% confidence intervals always including expected values for pigs of this age.[9] Even though there was a certain degree of variation in the mortalities observed in that period, overall mean mortality for both nursery and WF pigs was below 5%, and in more than 95% of the batches the mortality was below 10%. The impact of PED on the mortality of the first positive weaned batches was evident, since more than half of the 18 case batches having mortalities above 10% (Table 1). Use of mortality as an indicator of performance of growing pigs is impaired by the limited number of studies focused on defining baseline levels in grow-finish pigs.[10] Indeed, the variation observed in the batches produced before the PEDv outbreak in the flows under study likely reflects the impact of other variables (such as coinfection with other pathogens, or certain stressors or management practices) not captured in the analysis. Still, the absence of a visible trend in the mortality in a four month period supports the suitability of the selection of the control pair among batches produced within those 120 days, as all were considered to belong to the same population. Even with the limited sample size analyzed here, differences in mortality evidenced by the comparison of the paired values were enough to reach statistical significance, with a mean increase of at least 10% and 13 of 18 pairs displaying a difference above 5% (Table 1, Fig. 1). Three of the four pairs in which the control batch was weaned between 60–120 days before the infected batch displayed smaller differences than the rest of the data points (Fig. 1). Two of these flows had weaned other pigs between the infected and control batches that had been commingled with pigs from other sow farms, with mortality rates in these “intermediate batches” above 10% in all batches. However they could not be included in this study due to the lack of a suitable control pair for them (animals from the same sow farms that were PED negative within the previous 14–120 days). Therefore, our results would suggest that mortality in batches that were weaned several weeks after PEDv infected the sow farm was more similar to pre-PEDv mortality, and that the increase due to PEDv in the first pigs weaned after the outbreak may be even higher than estimated here.

Use of paired batches with the same origin allowed for control of certain factors known to affect mortality in growing pigs that occur at an early stage of their lives (such as mean weaning age). Such analytical procedure increased the homogeneity of the compared infected- and control-populations, in terms of breed, management and disease prevention procedures at the sow farm, thus accounting for their effect as possible confounders affecting mortality.[10,11] Data from WF and nursery batches were analyzed separately, but the fact that the period on feed for most of the WF pigs (only three PED- and one PED+ batch stayed >150 days) indicated that the study period in which performance was measured was very similar regardless of productive type of the site in which pigs were placed. Still, data were presented separately for both sites due to other management related factors that may differentiate WF and nursery sites, such as size of barns, floor, feeders, and that could have an impact, as suggested by the differences observed in the pre-PEDv batches). Infection with other pathogens could also increase mortality, and PRRSv was detected in four of the 18 flows before the PED outbreak, and in four case batches compared with one control batch. Still, among those three pairs in which only the case batch was positive (flows O, P and Q), difference in case-control mortality was less than 3% in two cases (Table 1), suggesting that PRRS was not a significant factor in the overall trend observed across flows. Moreover, the mean difference between the mortality recorded in case and control batches from the four flows affected with PRRS within the four months before the PED outbreak was lower than that observed in flows in which PRRSv was not detected throughout the whole study (8.7% and 14.2%, respectively).

The site where pigs were placed was not taken into account in the analysis due to the extensive variability found in the study population (that did not allow for any adjustment there). The location in which the pigs stayed during the period in which mortality was measured would undoubtedly influence this parameter. However, provided that weaned pigs were shipped to the different sites depending on the production needs and capacities and not depending on the PED status of the sow farm at that time, the “site effect” likely affected infected- and control-batches similarly, and for that reason, the authors do not think that such effect has affected the results reported here. As with any other observational study, causality cannot be demonstrated and for that reason, it is unclear if the higher mortality in PED+ batches was due to PEDv infection alone. However, we are not aware of any epidemiological factor that could increase mortality and that was significantly more prevalent in PED+ batches compared with PED- batches. For this reason, we believe that the most biologically sound explanation for the significantly poorer performance observed in PED+ batches was the infection with the virus.

Mortality can be directly recorded by swine barn managers and is related with profitability of swine operations.[10] This makes it an attractive target to evaluate the importance of PEDv in growing pigs. However, ADG. ADFI and FCR are also basic indicators of performance usually recorded in growing pigs.[7] Our data reveal that ADG was significantly lower in PED-positive compared with PED-negative batches, whereas ADFI remained unaltered (Table 1), thus yielding a mean increase in the FCR of 0.55. Assuming that the feed costs associated to produce a 40 lb feeder pig is 11.47$/head (mean estimate for December 2013–June 2014, [12]), our results would suggest a mean increase of the cost of each lb. of pig of around 16 cents due to the increased feed costs only. Variability in these parameters within the four months before the outbreak was larger than in the case of mortality, as shown for FCR in Fig. 1, therefore suggesting that these results should be interpreted carefully given the limited sample size. Still, comparison of paired values revealed a consistent trend towards a poorer performance of pigs from case batches (Fig. 3a and 3b). This suggests that while clinical signs of diarrhea can be mild in growing pigs, the negative consequence on performance is important. In summary, our results demonstrate that mortality, ADG and FCR recorded in growing pigs (mean ages 23–90 days) were significantly poorer from those observed in pigs weaned 14 to 120 days before the introduction of PED. This is the first reported description of the effect of PEDv infection in performance of growing pigs that survive the infection at the sow farm.

## Supporting Information

S1 Dataset Pig batches produced by the 18 flows that broke with PED during the study period (March 2013-June 2014) in the 120 days before the first PED+ batch. (XLS)
